# Physiological responses to folate overproduction in *Lactobacillus plantarum *WCFS1

**DOI:** 10.1186/1475-2859-9-100

**Published:** 2010-12-17

**Authors:** Arno Wegkamp, Astrid E Mars, Magda Faijes, Douwe Molenaar, Ric CH de Vos, Sebastian MJ Klaus, Andrew D Hanson, Willem M de Vos, Eddy J Smid

**Affiliations:** 1 TI Food & Nutrition, Wageningen, Nieuwe Kanaal 9A, 6709 PA, Wageningen, The Netherlands; 2 NIZO food research, Kernhemseweg 2, P.O. Box 20, 6710 BA, Ede, The Netherlands; 3 Plant Research International, Wageningen-UR, P.O. Box 16, 6700AA, Wageningen, The Netherlands; 4 Horticultural Sciences Department, University of Florida, Gainesville, Florida 32611, USA; 5 Institut Químic de Sarrià, Universitat Ramon Llull, 08017, Barcelona, Spain; 6 Agrotechnology & Food Sciences group, P.O. Box 17, 6700 AA Wageningen, The Netherlands; 7 Laboratory of Microbiology, Wageningen University, Dreijenplein 10, 6703 HB Wageningen, The Netherlands; 8 Laboratory of Food Microbiology, Wageningen University, Bomenweg 2, P.O. Box 8129, 6700 EV Wageningen, The Netherlands; 9 Securetec Detektions-Systeme AG, Eugen-Sänger-Ring 1, 85649 Brunnthal, Germany

## Abstract

**Background:**

Using a functional genomics approach we addressed the impact of folate overproduction on metabolite formation and gene expression in *Lactobacillus plantarum *WCFS1. We focused specifically on the mechanism that reduces growth rates in folate-overproducing cells.

**Results:**

Metabolite formation and gene expression were determined in a folate-overproducing- and wild-type strain. Differential metabolomics analysis of intracellular metabolite pools indicated that the pool sizes of 18 metabolites differed significantly between these strains. The gene expression profile was determined for both strains in pH-regulated chemostat culture and batch culture. Apart from the expected overexpression of the 6 genes of the folate gene cluster, no other genes were found to be differentially expressed both in continuous and batch cultures. The discrepancy between the low transcriptome and metabolome response and the 25% growth rate reduction of the folate overproducing strain was further investigated. Folate production per se could be ruled out as a contributing factor, since in the absence of folate production the growth rate of the overproducer was also reduced by 25%. The higher metabolic costs for DNA and RNA biosynthesis in the folate overproducing strain were also ruled out. However, it was demonstrated that folate-specific mRNAs and proteins constitute 8% and 4% of the total mRNA and protein pool, respectively.

**Conclusion:**

Folate overproduction leads to very little change in metabolite levels or overall transcript profile, while at the same time the growth rate is reduced drastically. This shows that *Lactobacillus plantarum *WCFS1 is unable to respond to this growth rate reduction, most likely because the growth-related transcripts and proteins are diluted by the enormous amount of gratuitous folate-related transcripts and proteins.

## Background

Microorganisms are often used as cell factories to produce a wide range of metabolites and proteins. Metabolic engineering is a suitable method to increase the production levels of these desired compounds. Feasibility studies with lactic acid bacteria have been performed in which strains were constructed with increased production of metabolites such as D-alanine, sorbitol, riboflavin, and folate [1-4]. In *Lactococcus lactis*, overproduction of alanine dehydrogenase in a lactate dehydrogenase (LDH) deficient strain resulted rerouting the glycolytic flux towards alanine [3]. In another case, overexpression of the complete riboflavin gene cluster in *L. lactis *resulted in a high riboflavin producing *L. lactis *strain [2]. A third example is the combined overexpression of the folate gene cluster and the *p*-aminobenzoate (*p*ABA) gene cluster in *L. lactis *which resulted in a high folate producing strain [1]. The latter strain was able to produce 100-fold more folate (total folate levels) when compared to control strains. Folate biosynthesis proceeds via the conversion of GTP in seven consecutive steps towards the biologically active cofactor tetrahydrofolate (THF). The biosynthesis of THF includes two condensation reactions. The first is the condensation of *p*ABA with 2-amino-4-hydroxy-6-hydroxymethyl-7,8-dihydropteridine to produce dihydropteroate. Subsequently, glutamate is attached to dihydropteroate to form dihydrofolate [5]. Without *p*ABA, no THF can be produced and THF is needed as the donor and acceptor of one-carbon groups (i.e methyl, formyl, methenyl and methylene) in the biosynthesis of purines and pyrimidines, formyl-methionyl tRNA^fmet ^and some amino acids [6,7].

The model organism *Escherichia coli *is commonly used for recombinant overexpression of proteins [8]. This micro-organism has a long history of application in the production of a vast range of proteins such as insulin, human growth hormones or interferon [9-11]. A problem with overexpression of recombinant or homologous proteins on high-copy plasmids is that the desired phenotype may be rapidly lost when propagated for prolonged periods of time [12]. One cause for this instability is a metabolic burden [13,14]. In *E. coli*, for example, the overproduction of a truncated elongation factor EF-*Tu *leads to a reduced growth rate of the strain [15]. It is evident that this EF-*Tu *overproducing strain is handicapped because of the production of a non-functional protein. In this case the production of functional proteins is reduced since the functional and non-functional proteins compete for the same resources of the translation machinery.

Lactobacilli are commonly used to ferment food products like meat, vegetables and dairy products [16]. *Lactobacillus plantarum *is a well-characterized lactic acid bacterium and strain WCFS1 was the first in the genus *Lactobacillus *for which the entire genome sequence became publicly available [17]. Previously, a high folate-producing *L. plantarum *WCFS1 strain was constructed that produced more than 100-fold increased folate pools, when compared to the control strain. Remarkably, this strain exhibited a 20-25% reduction in growth rate [18].

It remains unclear whether high production of specific secondary metabolites such as folate can provoke a large cellular response. This paper describes the impact of metabolic engineering of folate production on the overall performance of the cell. Functional genomics tools, including transcriptomics and metabolomics, were used to elucidate global effects of folate overproduction. Leads from this analysis were used to help explain the growth rate reduction upon the overexpression of the folate gene cluster.

## Results

### Metabolite formation upon folate overproduction

**Figure 1:**
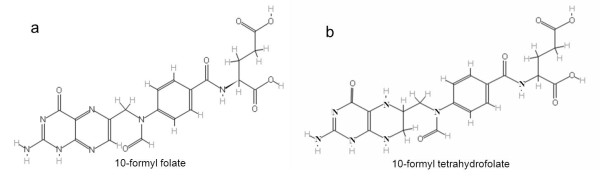
**The structure of 10-formyl folate (a) and 10-formyl tetrahydrofolate (b)**.

First of all, the impact of folate overproduction on metabolite formation and the transcript profile was determined. Secondly, specific analyses were performed to determine mechanisms that cause the observed growth rate reduction upon folate overproduction. Previously it was shown that homologous overexpression of the folate gene cluster of *L. plantarum *results in high folate pools [18]. It was shown that there is 55-fold more folate produced in *L. plantarum *cultures harboring plasmid pNZ7026 (which carries all genes in the folate biosynthesis pathway) when compared to the control strain carrying plasmid pNZ7021 (empty expression vector) [18]. Using differential metabolomics it was determined whether specific metabolites were more or less abundant in *L. plantarum *harboring pNZ7026 in comparison to *L. plantarum *carrying the control plasmid pNZ7021. Both strains were cultivated in a pH controlled chemostat culture in the presence of *p*ABA. At steady state, cells were harvested, quenched and extracted for metabolome analysis by LC-MS/MS. In total 18 metabolites with differential abundance were detected (Table 1). Of this group, 15 metabolites were significantly more abundant in *L. plantarum *harboring pNZ7026 and 3 metabolites were significantly less abundant. Five of the 15 metabolites, that were more abundant in *L. plantarum *harboring pNZ7026, could be linked directly to folate biosynthesis. The metabolite assigned as 10-formyl folate (Figure 1a) showed the largest difference in relative abundance; this molecule was 117-fold more abundant in *L. plantarum *harboring pNZ7026 as compared to the control strain (pNZ7021). We also detected a 33- and 2.1-fold increase in abundance of a 10-formyl folate isomer and 10-formyl tetrahydrofolate (Figure 1b), respectively. One metabolite, 2-amino-1,4-dihydro-4-oxo-6-pteridinecarboxylic methyl ester, is a known breakdown product of folate. When folate is exposed to light it decomposes into the latter compound and 2-amino-4-hydroxypteridine [19]. The other 11 metabolites cannot be linked directly to the folate biosynthesis pathway and their involvement remains to be investigated. Only 3 metabolites were present in a significantly lower abundance (less than 2-fold) in *L. plantarum *harboring pNZ7026; these components were putatively annotated as thymidine, 3-dehydroshikimate and 1-amino guanosine. In conclusion, the overexpression of the folate gene cluster leads to a massive accumulation in 10-formyl folate and other folate related metabolites. However, the global impact of folate overproduction on metabolite accumulation is relatively low with only 18 metabolites showing a significantly different relative abundance. In addition, folate and pterin (intermediates in the folate pathway) pools were analyzed by a microbiological assay and HPLC in the intra- and extracellular fractions, respectively (Table 2). High intracellular pterin pools were detected only in *L. plantarum *harboring pNZ7026 in the absence of *p*ABA. The principal pterin was identified as 6-hydroxymethylpterin from its chromatographic properties, and was detected in the extra- and intracellular fraction. In the folate biosynthesis pathway, 6-hydroxymethylpterin (in its dihydroform) is activated by pyrophosphorylation and then condensed with *p*ABA to form dihydropteroate, which is then glutamylated to yield folate. This demonstrates that *L. plantarum *WCFS1 cannot convert 6-hydroxymethylpterin into folate in a medium lacking *p*ABA. In addition, Table 2 shows that independent from the presence of *p*ABA in CDM; the growth of *L. plantarum *harboring pNZ7026 was 25% lower when compared to control strain. In summary, the high folate or high pterin levels alone cannot explain the growth rate reduction of the folate overproducing strain.

**Table 1:** Metabolites that differ significantly in relative abundance between L. plantarum WCFS1 harboring pNZ7026 and pNZ7021

Putative compound name	ratio pNZ7026/pNZ7021	apparent mass [M+H]^+^	Ppm Δ mass
10-formyl folate	117.2	470.1431	2.6
10-formyl folate isomer	33.6	470.1493	15.8
Novel C_17_H_14_O_3_	20.4	267.1007	-5.3
Novel folate C_24_H_23_N_7_O_5_	19.4	490.1796	-7.9
1-[(2-methoxyphenyl)methyl]-5-nitro-2H-indazol-3-one	11.6	300.1000	7.1
C_20_H_22_N_5_O_2_S	5.7	396.1491	0.3
Unidentified	5.7	728.2331	
2-amino-1,4-dihydro-4-oxo-6-pteridinecarboxylic methyl ester	4.9	222.0674	23.5
Unidentified	3.8	254.0952	
Adenosine	2.8	268.1066	-2.8
C_18_H_32_O_16_	2.6	505.1852	17.4
5-methylthioadenosine	2.4	298.1034	10.9
C_4_H_10_N_4_OS	2.2	163.0651	1.09
C_12_H_27_N_7_O_14_P_2 _e.g. nicotinamide arabinoside adenine dinucleotide	2.1	556.116	12.8
10-formyltetrahydrofolate	2.1	474.1813	7.3
Thymidine	0.6	243.0938	9.4
C_10_H_14_N_6_O_5 _e.g. 1-amino guanosine	0.6	299.1132	15.1
3-dehydroshikimate	0.5	173.0471	14.3

The table shows the putative compound name, the relative abundance of the metabolite, apparent mass of the compound, and the deviation of the apparent mass compared to the expected mass.

**Table 2:** Intracellular and extracellular concentration of 6-hydroxymethylpterin and folate in L. plantarum harboring pNZ7021 and pNZ7026 in the presence and absence of pABA

		6-Hydroxymethylpterin (nmol/50-ml culture)		Folate μg/L per OD_600 _unit	
*L. plantarum *harboring	μ_max _h^-1^	Intracellular	Extracellular	Intracellular	Extracellular
pNZ7021	0.61 (0.02)	0.1	ND^a^	ND	ND
pNZ7021 + *p*ABA	0.60 (0.02)	0.1	ND^a^	3.93 (1)	8.56 (3)
pNZ7026	0.45 0.02)	3.0	1692	ND	ND
pNZ7026 + *p*ABA	0.44 (0.01)	0.2	217	216 (29)	3020 (202)

^a ^ND, not detectable

Note: The standard deviation of the folate assay is shown between brackets.

### Transcriptional profiling of folate overproducing cells

DNA microarrays were used to analyze differential gene expression in response to high intracellular folate pools. For this study, we selected two different cultivation conditions (continuous and batch culture) to make a distinction between gene expression profiles specific for high folate pools and secondary effects of the overexpression of the folate gene cluster, e.g. differences in growth rate (as can be observed in batch cultures Table 2). It is assumed that any similarity in gene expression between both cultivation conditions is due to the production of folate or the high folate pools. All genes which are significantly up- or down-regulated are presented in Table 3. The only genes that were differentially expressed both in batch and continuous culture are the 6 genes of the folate biosynthesis cluster (shown in bold and italics in Table 3). Because these genes were constitutively overexpressed on a high copy plasmid, the observed response is expected. This analysis shows that high folate pools or the elevated synthesis of folate does not lead to a global transcriptional response. Instead, it was found that 8 and 11 other genes responded specifically to secondary effects of the overexpression of the folate cluster in continuous and batch culture, respectively (Table 3). In continuous culture the 8 differentially expressed genes are involved in cation uptake or belong to a cell surface cluster which is predicted to be involved in the uptake of complex carbohydrates [20]. The biological relevance of down-regulation of these genes is unclear. In the batch experiment a total of 11 genes were significantly regulated upon the overexpression of the folate gene cluster. One gene cluster, involved in pyrimidine biosynthesis, appears to respond specifically to the growth rate reduction; as was noted in Table 2. Remarkably, this gene cluster was also down-regulated when the folate gene cluster was overexpressed in the absence of *p*ABA (data not shown). The pyrimidine biosynthesis gene cluster is composed of 9 genes, from lp_2697 (*pyrE*) until lp_2704 (*pyrR1*), including a gene upstream of the pyrimidine gene cluster, lp_2696 and a pyrimidine transporter *pyrP*, lp_2371. Two additional genes, *ansB *and *rhe1*, are up-regulated upon the overexpression of the folate gene cluster in batch culture. AnsB (E.C. 3.5.1.1) is involved in the conversion of L-aspargine into L-aspartate. Rhe1 is involved in the unwinding of RNA-helices. The biological relevance of the differential expression of these genes under those conditions remains unclear. However, from these experiments it can be concluded that the reduced growth rate (as observed in batch culture in the presence and absence of *p*ABA; Table 2) does not trigger a large transcriptional response, instead only a few genes could potentially be linked to the growth rate reduction. Moreover, none of the genes of *L. plantarum *appears to respond specifically to high folate pools, or the increased biosynthesis of folate.

**Table 3:** Overview of genes that are differentially expressed in the L. plantarum strain harboring pNZ7026 when compared to the control strain (pNZ7021)

		Continuous culture		Batch culture	
Synonyms	Sub class	log2 ratio	**Holmes sign**.	log2 ratio	**Holmes sign**.
*rhe1*	ATP dependent RNA helicase	-0.38	1.00	-1.59	0.07
*mtsC*	Cations	1.33	0.00	-0.01	1.00
*mtsB*	Cations	1.43	0.00	-0.02	1.00
*mtsA*	Cations	1.46	0.00	-0.07	1.00
*pyrP*	Nucleoside, purines and pyrimidines	0.19	1.00	1.89	0.02
*Lp_2696*	Conserved: membrane proteins	0.04	1.00	1.32	0.07
*pyre*	Pyrimidine ribonucleotide biosynthesis	0.15	1.00	2.84	0.07
*pyrF*	Pyrimidine ribonucleotide biosynthesis	0.14	1.00	2.73	0.01
*pyrD*	Pyrimidine ribonucleotide biosynthesis	0.14	1.00	2.75	0.00
*pyrAB*	Pyrimidine ribonucleotide biosynthesis	0.11	1.00	2.74	0.00
*pyrC*	Pyrimidine ribonucleotide biosynthesis	0.14	1.00	3.04	0.00
*pyrB*	Pyrimidine ribonucleotide biosynthesis	0.08	1.00	2.88	0.00
*pyrR1*	Other	-0.06	1.00	2.01	0.00
*ansB*	Glutamate familiy	-0.16	1.00	-1.37	0.08
*mntH2*	Cations	1.28	0.00	0.45	1.00
*folP*	Folate biosynthesis	***-5.50***	0.00	***-5.86***	0.00
*Xtp2*	Folate biosynthesis	***-5.31***	0.00	***-6.28***	0.00
*folC2*	Folate biosynthesis	***-5.85***	0.00	***-6.44***	0.00
*folE*	Folate biosynthesis	***-5.36***	0.00	***-6.41***	0.00
*folK*	Folate biosynthesis	***-5.80***	0.00	***-6.47***	0.00
*folB*	Folate biosynthesis	***-5.54***	0.00	***-6.21***	0.00
*Lp_3412*	Cell surface proteins: other	-1.53	0.01	-0.28	1.00
*Lp_3413*	Cell surface proteins: other	-1.93	0.00	-0.37	1.00
*Lp_3414*	Cell surface proteins: other	-2.10	0.00	-0.43	1.00
*Lp_3415*	Other	-1.17	0.00	-0.72	0.72

### Mechanism of growth rate reduction

Functional genomics tools such as transcriptomics and metabolomics showed that folate overproduction in *L. plantarum *has a low impact on the global transcription profile and metabolite formation. The growth rate of *L. plantarum *harboring pNZ7026 was reduced by 25%, when compared to *L. plantarum *harboring pNZ7021 in the presence or absence of *p*ABA (Table 2). This notion shows that a high folate pool itself cannot explain the growth rate reduction. To get insight into potential mechanisms for the growth rate reduction we explored several possible causes of reduced growth rate: i) metabolic costs for mRNA synthesis and plasmid synthesis; ii) increased pools of mRNA and/or protein of the transcription/translation machinery; and iii) depletion of GTP by its drainage away for folate production. The experimental approaches to investigate the involvement of these mechanisms are described below.

### Effect of elevated mRNA synthesis and plasmid replication on the growth rate

It was determined whether the growth rate reduction could be explained by increased metabolic cost for mRNA synthesis or plasmid replication. When comparing the signals of all transcripts (9606 gene related probes representing the 3688 genes) on the microarrays with the signals of the folate biosynthesis transcripts (a total of 18 probes on the microarray), it was found that the latter are the highest expressed genes on the entire microarray, even higher than glycolytic and ribosomal protein transcripts. In *L. plantarum *WCFS1 harboring pNZ7021 and pNZ7026 the folate mRNAs are on average 0.16% and 8.3% of the total mRNA pool, respectively. Next, it was investigated whether the cost for mRNA synthesis could explain the reduced growth rate of *L. plantarum *harboring pNZ7026. Simultaneously, the difference in plasmid size of pNZ7021 and pNZ7026, with 3.3 and 7.7 Kb, respectively, was also marked as a potential cause, reflecting the plasmid replication cost and assuming a similar copy number for both plasmids. To test this explanation, the growth performance, mRNA synthesis and plasmid copy numbers were determined for *L. plantarum *harboring pNZ8148 (empty vector), pNZ7030 (folate gene cluster in sense orientation) and pNZ7031 (folate gene cluster in antisense orientation). The gene expression using plasmids pNZ7021 and pNZ7026 is constitutive which is in contrast to pNZ8148, pNZ7030 and pNZ7031, in these plasmids gene expression is regulated by the addition of nisin. Using the strains with the latter plasmids we were able to make a distinction between the effect of mRNA synthesis alone (*L. plantarum *harboring pNZ7031) and the combined effects of mRNA and protein synthesis (*L. plantarum *harboring pNZ7030). *In silico *analysis using MEME and MAST predicted no putative functional ribosome binding sites on the folate gene cluster in the antisense orientation (pNZ7031), showing that no antisense-proteins are likely to be made using this construct. Growth rates and folate pools were determined in the strains carrying the different plasmids (Table 4). The growth rate of *L. plantarum *harboring pNZ7030 was reduced regardless of whether gene expression was induced with nisin. The growth rates of *L. plantarum *containing pNZ8148 (control plasmid) and pNZ7031 (antisense orientated plasmid) were unaffected. Interestingly, overexpression of the folate gene cluster in the antisense orientation results in a 6-fold increase in folate production pools when compared to control strain. By RT-qPCR it was confirmed that *L. plantarum *strains harboring pNZ7030 and pNZ7031 produced the anticipated mRNAs (Table 5). The relative expression level in *L. plantarum *harboring pNZ8148 is arbitrarily set at 1 and the gene expression values in the two other strains were related to this strain. Overexpression of the folate genes in the sense and antisense orientations resulted in a vast increase in the expected mRNAs, but only in *L. plantarum *harboring pNZ7030 was a reduced growth rate observed, suggesting that mRNA production itself is not responsible for the growth impairment. The relative plasmid copy number of *L. plantarum *harboring pNZ8148, pNZ7030 and pNZ7031 before and after nisin induction is shown in Table 6. This analysis shows that the relative plasmid copy number varies between the different constructs. The strain with the highest plasmid copy number is *L. plantarum *harboring pNZ7030, suggesting that increased plasmid synthesis could explain the growth rate reduction. However, a 5-fold increase in relative copy number for *L. plantarum *harboring pNZ7031 in the induced and uninduced condition did not result in a growth rate reduction, showing that relative copy numbers may vary between strains and are not necessary linked to growth rate effects. In conclusion, the observed growth rate reduction in the folate overproducer cannot be attributed to the increased metabolic costs for mRNA synthesis or plasmid replication.

**Table 4:** Growth rates, and folate pools in the uninduced and induced cell culture of L. plantarum harboring pNZ8148, pNZ7030, and pNZ7030

*L. plantarum*	0 ng/ml nisin		25 ng/ml nisin	
**Harboring**	**Folate μg/L per OD_600 _unit**	**μ_max _h^-1^**	**Folate μg/L per OD_600 _unit**	**μ_max _h^-1^**
pNZ8148	6 (0.6)	0.40 (0.04)	6 (0.4)	0.369 (0.01)
pNZ7030	783. (63)	0.31 (0.02)	1736 (211)	0.24 (0.03)
pNZ7031	35 (3)	0.41 (0.02)	31 (4)	0.44 (0.01)

Note; standard deviation is given in parentheses.

**Table 5:** Relative expression of folB and folP in L. plantarum harboring pNZ8148, pNZ7030, and pNZ7030 after 20 minutes and 4 hours following nisin induction and in the uninduced cultures

		0 ng/ml nisin	25 ng/ml nisin
time minutes	*L. plantarum *harboring	Average expression *folB*-*folP*	Average expression *folB*-*folP*
20	pNZ8148 sense	1	1
20	pNZ7030 sense	64	584
20	pNZ7031 antisense	84	2864
240	pNZ8148 sense	1	1
240	pNZ7030 sense	1	38
240	pNZ7031 antisense	3	11

Expression values of the two folate genes, folB and folP, are normalized to groES, and are indicated as average expression folB-folP.

**Table 6:** Relative copy number for pNZ8148, pNZ7030, and pNZ7030 in L. plantarum determined in the induced and uninduced cultures

	0 ng/ml nisin	25 ng/ml nisin
*L. plantarum *harboring	Relative copy number	Relative copy number
pNZ8148	218 (2)	228 (17)
pNZ7030	2245 (197)	801 (51)
pNZ7031	2058 (171)	387 (17)

Note: The standard deviation is presented between brackets, and is calculated from two independent measurements.

### Analysis of mRNA and protein pools upon overexpression of the folate gene cluster

Another explanation for the growth rate reduction of the folate overproducing strain might be competition between growth related and gratuitous transcripts/proteins for the transcription/translation machinery. It was described above that in *L. plantarum *WCFS1 harboring pNZ7026, the transcripts derived from the folate genes constitute 8.3% of the total mRNA pool. Since the growth rate of *L. plantarum *harboring pNZ7030 was also reduced, the same analysis was performed on the mRNA pools of this strain. It was determined that the folate specific mRNA pool in this strain constitute an impressive 33% of the total mRNA pool. Consequently, the overexpression of the folate gene cluster results in an enormous accumulation of folate specific mRNAs.

**Figure 2:**
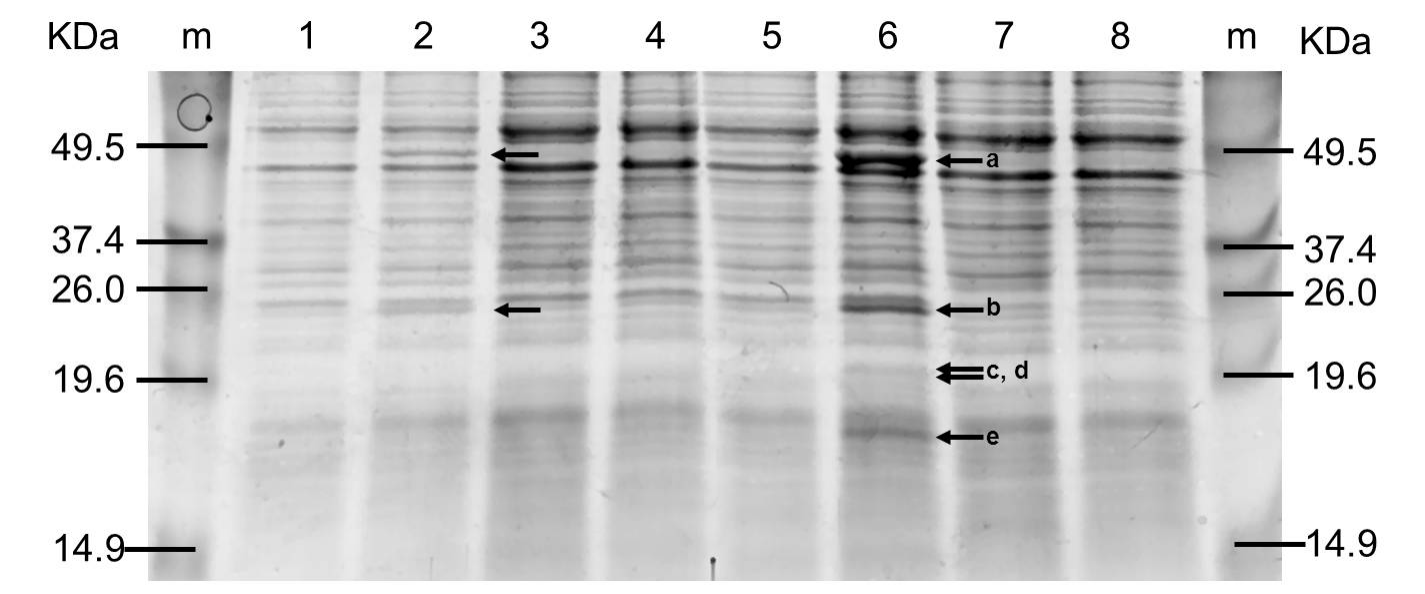
**SDS-PAGE gel showing a standard protein marker with the indicated molecular weights (in kDa) on both outside lanes of the gel**. Lane 1 till 8 show the protein content of *L. plantarum *harboring pNZ7021, pNZ7026, pNZ8148 (0 ng/ml nisin), pNZ8148 (25 ng/ml nisin), pNZ7030 (0 ng/ml nisin), pNZ7030 (25 ng/ml nisin), pNZ7031 (0 ng/ml nisin) and pNZ7031 (25 ng/ml nisin), respectively. In lane 6 five bands are indicated as: a) (FolC2, 50.4 KDa), b) (FolP, 29.2 KDa), c) (Xtp2, 21.7 KDa), d) (FolE, 21.0 KDa), and e) (FolK, 18.9 KDa). In lane 2 the bands a) (Folc2, 50.4 KDa) and b) (Folp, 29.2 KDa) were detected.

Also, the relative abundance of the folate biosynthesis enzymes was determined by SDS-PAGE for *L. plantarum *WCFS1 harboring pNZ7021, pNZ7026, pNZ8148, pNZ7030, and pNZ7031 (in pNZ8148, pNZ7030, and pNZ7031 with and without induction with nisin) (Figure 2). The protein band patterns on the SDS-PAGE gel were quantified using ImageJ. The total peak area (representing the total protein content) and the peak area of folate biosynthesis proteins were determined. Clear folate protein peaks could be distinguished for *L. plantarum *harboring pNZ7030 that matched with the expected protein sizes (5 of the 6 proteins were detected, 1 protein is too small for detection on gel). For *L. plantarum *harboring pNZ7026, the two largest proteins were identified (Figure 2). The folate protein content in *L. plantarum *harboring pNZ7021, pNZ8148 and pNZ7031 were set at 0% folate proteins. In *L. plantarum *containing pNZ7026 and pNZ7030 (after nisin induction) the folate proteins constitute 4 and 10% of the total protein pool, respectively. The relatively high production of folate related transcripts and proteins in relation to transcripts and protein needed for growth, indicates that the metabolic burden of folate overproduction is an important factor.

### The drain on GTP pools by folate production

Apart from being a precursor in folate biosynthesis, GTP is also consumed during the synthesis of DNA and RNA. The drain on the GTP pool due to excessive folate production is calculated for *L. plantarum *WCFS1 harboring pNZ7026. Based on the biomass composition of *L. plantarum *WCFS1 [21], it was determined that 0.10 mmol/g dry weight (DW) GTP is stored in DNA and RNA. In *L. plantarum *harboring pNZ7026 approximately 0.04 mmol/g DW GTP is stored in folate. Assuming a free GTP pool of approximately 0.5 mM [22] and an internal bacterial cell volume of 3.6 μl/mg protein [23], the free GTP pool is calculated to be in the order of magnitude of 10^-6 ^mol/g DW and therefore negligible. Based on these numbers it was estimated that 29% of the GTP in *L. plantarum *harboring pNZ7026 is directed into folate (or pterins). For *L. plantarum *harboring pNZ7021 this is less than 0.03%. Surprisingly, the large drain on GTP did not provoke a transcriptional response with respect to expression of purine biosynthesis genes in *L. plantarum *harboring pNZ7026. These calculations show that folate overproduction may impose a large drain on the biosynthesis of important molecule such as GTP, without affecting the expression of genes related to purine biosynthesis.

## Discussion

Overexpression of the folate gene cluster in *L. plantarum *leads a high level of folate production, but this is also accompanied by a reduction in growth rate. This reduction, however, did not provoke a clear transcriptional or metabolic response. This is in contrast to *Saccharomyces cerevisiae *and *Escherichia coli *where gene expression profiles were found to be profoundly different at varying growth rates [24,25]. It appears that the folate overproducing *L. plantarum *strain is unable to respond to the growth rate reduction. Our experiments demonstrated that the folate specific mRNAs constitute 8.3% and 33% of the total mRNA pool of the cell in cells using the constitutive- (pNZ7026) and nisin inducible plasmid (pNZ7030), respectively. These mRNA levels were even higher than glycolytic- and ribosomal protein transcripts. Based on the observed inability of the cell to respond to the imposed growth rate reduction, we hypothesize that the reduced growth rate in the overproducer is caused by the high proportion of gratuitous transcripts which dilute all growth related mRNAs (such as those for ribosomal protein synthesis). This is not trivial since the growth rate itself is largely dictated by the level of protein synthesis and RNA production [26]. Additionally, it is reported that at a high growth rate the mRNAs become ever more crowded with ribosomes, thereby the average spacing of ribosomes on the mRNA shifts from 120 to 60 nucleotides at higher growth rates [27]. When a huge number of ribosomes start to occupy gratuitous mRNAs (such as folate mRNAs), translation of growth related mRNAs (such as ribosomal proteins themselves) will be reduced. In many cases growth rate reductions upon the overexpression of gratuitous proteins have been referred to as a metabolic burden, and have been associated with the production of specific proteins which lead to a reduction in growth rate [15,28]. However, since in bacteria the process of transcription and translation are tightly coupled, it might very well be that dilution of growth related mRNAs is crucial for explaining the growth rate reduction upon overexpression. Still, the need for rare tRNAs cannot be excluded as one of the factors explaining the growth rate reduction. It was found that three codons (tRNA^Arg ^(AGG), tRNA^Cys^, (UGC), and tRNA^Ile ^(AUA)) were 5-fold less abundant in the genome of *L. plantarum *WCFS1 when compared to the sequence of the folate gene cluster (unpublished data). In *E. coli *it was observed that the overexpression of tryptophanase (EC 4.1.99.1) resulted in a growth rate reduction mainly because it led to a shortage of a specific tRNA molecules [29].

The reduced growth rate of *L. plantarum *harboring pNZ7026 suggests a kind of stress, but besides the down-regulation of pyrimidine gene cluster (in the batch cultures) no generic stress response was provoked. Applying stress to a microorganism often leads to slower growth. In *E. coli*, for example, the transcriptional response was determined in a strain carrying a plasmid for overproduction of chloramphenicol acetyltransferase in comparison with a wild-type strain carrying no recombinant plasmids [14]. From this experiment it was evident that overproduction of chloramphenicol acetyltransferase provoked stress to the cell, as indicated by the large number of stress-response and growth related genes that were differentially expressed. The response of *L. plantarum *to folate overproduction is clearly different from the response of *E. coli *towards overproduction of chloramphenicol acetyltransferase. One possible explanation is that we have used a control strain carrying an empty plasmid, and therefore both the control strain and the overproducer experience the presence of chlorampenicol.

The metabolomics data in our study indicate that only a few metabolites were significantly affected in their relative abundance in *L. plantarum *harboring pNZ7026. One metabolite, 10-formyl folate, was 117-fold more abundant in *L. plantarum *harboring pNZ7026. This was unexpected since it is assumed that the reduced derivative, 10-formyl tetrahydrofolate, is produced by the organism. In *L. lactis*, for example, 10-formyl tetrahydrofolate was detected as the most dominant type of folate [30]. Since tetrahydrofolate derivatives are known to be unstable [31-33] this component may have been converted to the oxidized form (folate) in the bacterial cells or during metabolite extraction or LC-MS analysis. The compound 10-formyl folate is supposed to be biologically inactive [34], however, we have demonstrated that 10-formyl folate can be used by the indicator strain in the microbiological folate assay.

Remarkably, overexpression of the folate gene cluster in the antisense orientation results in 6 fold increased folate production when compared to control strain. Possibly, the antisense mRNA stabilizes the sense mRNA. This partially double stranded RNA is expected to be protected from degradation by RNA nucleases which may explain increased folate production and consequently elevated folate pools. Such mechanism of antisense overexpression could be exploited as a novel procedure for overproduction of proteins or metabolites.

Based on our results, we calculated that approximately 29% of the synthesized GTP is directed into folate, indicating that the growth rate reduction is, at least partly, linked with a shortage in the supply of GTP. Therefore, since folate overproduction has a large drain on GTP pools, this might have implications for protein synthesis, since GTP hydrolysis for protein synthesis alone accounts for more than 32% of the total energy turnover a of lactic acid bacterium [35,36]. Transcriptome analysis showed no differential expression of the purine biosynthesis genes, suggesting that either there is no shortage in GTP supply, or GTP shortage does not provoke a transcriptional response to the purine genes. In *Bacillus subtilis*, a positive correlation was found between free GTP pools and the growth rate [37]. In *L. lactis*, the GMP-synthetase inhibitor, decoyinine, reduced the free GTP pool 2-fold, and reduced the growth rate of the organism [22]. When comparing the metabolome of the control strain with the folate overproducer, no reduction in relative abundance of GMP, GDP, or GTP was detected in our metabolome analysis. The only metabolite that could be linked to GTP shortage is 1-amino guanosine. However, it remains unclear whether this component can be phosphorylated, since few nucleoside kinases are known in lactic acid bacteria [36,38].

## Conclusion

High copy plasmids are often used for the overproduction of commercially interesting proteins or metabolites. In *Lactobacillus plantarum *WCFS1, homologous overexpression of entire gene cluster encoding folate biosynthsis results in high folate production. An important obstacle for robust folate production is the reduced growth rate of this overproducing strain. In the folate overproducing *L. plantarum *strain we did not observe large changes in transcript or metabolite formation. Apparently, *L. plantarum *does not adequately respond to the adverse (metabolic) effects of excessive high levels of folate biosynthesis. A possible explanation for the observed growth rate reduction is competition between highly abundant non-growth related mRNAs (of the folate biosynthesis pathway) and growth related (household) mRNAs at the level of the transcription/translation machinery. This explanation is generally applicable for all microbial cell factories employing high copy overexpression vectors.

## Methods

### Bacterial strains, media and culture conditions

*Lactobacillus plantarum *WCFS1 and derivatives thereof (Table 7 for the complete list of used strains and plasmids) were cultivated at 37°C on Chemically Defined Medium (CDM), as described before [39]. Unless stated otherwise, CDM is complete. In a number of specific batch culture experiments *p*ABA was omitted or added, thereby using a concentration of 10 mg/L. Precultivations of *L. plantarum *harboring pNZ7021 and pNZ7026 was performed in non-pH regulated batch cultures using 56 mM glucose as fermentable substrate. *L. plantarum *harboring pNZ7021 and pNZ7026 was also cultivated in a pH-regulated batch fermentor and in chemostat culture on CDM supplemented with 25 mM glucose. A concentration 80 mg/L chloramphenicol (CM) was used in batch and continuous culture. For the construction of genetically modified strains, MRS broth and agar was used (Difco, Surrey, U.K.). For selection on MRS plates 10 mg/L CM was applied to the agar. *Lactococcus lactis *was grown at 30°C on CDM supplemented with 56 mM glucose as described previously [40,41]. Transformed *L. lactis *strains were cultivated and selected on M17 broth [42] and agar using 10 mg/L CM.

**Table 7:** List of strains, constructed plasmids, and primers used in this study

Material	Relevant features	Source of reference
**Strains**		
*L. lactis*; NZ9000	MG1363 *pepN:nisRK*, Cloning host	[44]
*L. plantarum *WCFS1	Cloning host, genomic DNA isolation	[17]
*L. plantarum NZ7100*	WCFS1 *:nisRK*, Cloning host	[46]
**Plasmids**		
pNZ7021	Cm^R^, pNZ8148 derivative, nisin promoter replaced by pepN promoter	[1]
pNZ7026	Cm^R^, pNZ7021 derivative containing the *folB*, *folP*, *folK, folE*, *xtp2 *and *folC2 *gene cluster of *L. plantarum *WCFS1	[18]
pNZ8148	Cm^R^, nisin regulated promotor	[44]
pNZ7030	CmR, pNZ8148 derivative containing *folB*, *folP*, *folK*, *folE*, *xtp2 *and *folC2 *gene cluster of *L. plantarum *WCFS1 in the sense orientation	(this study)
pNZ7031	CmR, pNZ8148 derivative containing *folB*, *folP*, *folK*, *folE*, *xtp2 *and *folC2 *gene cluster of *L. plantarum *WCFS1 in the antisense orientation	(this study)
**Primers**		
LpfBnco-F	CTGGGATACCCATGGGCATGATTC	
LpfPkpn-R	CGTCAAAAGGTACCGGACTAATCATTATTCG	
pNis-F	TAGTCTTATAACTATACTGAC	
LpfB-R	CTTGCCATTCGGCGTCCCCTCCACCTCAATTTCC	
LpfBatg-F	ATGGGCATGATTCGAATTAATAATTTACG	
LpfP-xbatest	GAATTTAATTATTTGCGACGCCCAAT	
FQPCRfolBS	CCTATCGAAACCAAGGTTCAACA	
RQPCRfolBS	ACAAATTCATCGACCACGTTACG	
FQPCRfolBAS	TCAACTTGTATGAATGGGTCGTTACA	
RQPCRfolBAS	CGTTCACGAGACCATCAATTACG	
FQPCRFPS	CATTATTAACGATGTGAACGCCTTT	
RQPCRFPS	CGCGACTGTCAGCCATCAAT	
FQPCRfPAS	CTAACAGCGTAATCAATTGCTTGGT	
RQPCRfPAS	CTTAAGGGTGGCCGGATCA	
groES-fo(2)	CCCAAAGCGGTAAGGTTGTT	
groES-re(2)	CTTCACGCTGGGGTCAACTT	
pfk-fo1	TCCAGGGACGATCGATAATGA	
pfk-re1	GCTTGCACGTTGGTGTTGAAC	

### Construction of genetically engineered strains

Genomic DNA of *L. plantarum *WCFS1 was isolated using established procedures [43]. PCR was performed using PFX (Invitrogen, Breda, The Netherlands), applying PCR cycles of 94°C for 30 sec denaturation, 43°C for 30 sec for primer annealing, and 68°C for elongation (1 min per Kb). DNA ligation was performed using T4 DNA ligase (Invitrogen) by overnight incubation at 16°C. DNA fragments were mixed at a 5:1 insert:vector weight ratio. Two nisin inducible vectors were constructed, based on pNZ8148 [44]. In one vector the folate gene cluster of *L. plantarum *was cloned under the control of the nisin promoter in the sense orientation and, in the other, in the antisense orientation. The folate gene cluster was amplified in the sense orientation by PCR using LpfBnco-F and LpfPkpn-R as forward and reverse primers, respectively. Both primers were modified to introduce a restriction site for cloning of the DNA fragments (modified bases underlined in Table 7). The insertion plasmid pNZ8148 and the amplified DNA were digested with KpnI and NcoI. Both fragments were mixed and used for T4 DNA ligation. The DNA mix was transferred to *L. lactis *NZ9000 for transformation by electroporation, using established procedures [45]. The electroporated *L. lactis *suspension was plated and incubated for 40 h at 30°C. Chloramphenicol (CM) resistant colonies were checked for the presence of proper plasmids by PCR with pNis-F and LpfB-R as forward and reverse primer, respectively. Positive colonies were grown and plasmid DNA was extracted and then isolated using Jetstar columns (Genomed GmbH, Bad Oeynhausen, Germany). The restriction profile of the plasmid was determined; the plasmid with the proper restriction profile was named pNZ7030. The antisense vector was made by amplification of the folate gene cluster using, LpfBatg-F and LpfPkpn-R as the forward and reverse primers, respectively. The amplified linear fragment of DNA was digested with KpnI, and pNZ8148 was digested with KpnI and PmlI. The digested PCR product and digested plasmid were mixed and used for T4-DNA ligation. The DNA mix was transferred *L. lactis *NZ9000 for transformation as described above and plated on M17 plates with CM. After 40 h of growth, CM resistant colonies were checked for the presence of the correct plasmid by PCR; pNis-F and LpfP-xbatest were used as forward and reverse primer, respectively. Positive colonies were grown and plasmid DNA was extracted and then isolated using Jetstar columns. The restriction profile of the plasmid was determined and the plasmid with the proper restriction profile was named pNZ7031. The plasmids pNZ8148, pNZ7030 and pNZ7031 were used for transformation of *L. plantarum *NZ7100 [46] by electroporation using established procedures [47], and plated on MRS with CM. CM-resistant colonies were checked for the proper plasmid by PCR, using the primers as described above. Colonies with the proper plasmid were grown on CDM with the 80 mg/L CM and stored at -80°C in glycerol stocks waiting for further use.

### Continuous culture

Chemostat cultivation was performed in a 1-L reactor (Applikon Dependable Instruments, Schiedam, The Netherlands) containing 0.5 L CDM. Temperature was controlled at 37°C. *L. plantarum *harboring pNZ7021 and pNZ7026 were inoculated in the reactor; first exponential growth of the culture was allowed until the maximal turbidity at 600 nm was reached. Next, the dilution rate of both cultures was set at 0.25 h^-1^. Steady state was assumed after 5 volume changes. A stable pH of 5.5 was maintained by titration with 5 M NaOH, the pH was monitored by an ADI 1020 fermentation control unit (Applikon Dependable Instruments, Schiedam, The Netherlands). Anaerobic conditions were obtained by flushing the headspace of the reactor with nitrogen gas.

### Folate, *p*ABA and pterin analyses

Folate was quantified using the microbiological assay, including enzymatic deconjugation of polyglutamate tails [48,49]. Pterin pools were determined (after oxidation to the aromatic forms) by HPLC in the intracellular and extracellular fractions of *L. plantarum *WCFS1 cultures using the procedures described by Klaus [50]. The 6-hydroxymethylpterin standard for HPLC was purchased from Schircks (Jona, Switzerland).

### Transcriptome analysis

Cultures of *L. plantarum *WCFS1 strains were quenched using the cold methanol method [51]. Total RNA was isolated and extracted as described before [52]. The RNA concentration was determined with the ND-1000 spectrophotometer (NanoDrop Technologies Inc., USA). The quality of the isolated RNA was checked using the 2100 Bioanalyser (Agilent Technologies, Santa Clara, CA, USA); a ratio of 23 S over 16 S rRNA of ≥1.6 was taken as satisfactory. For cDNA synthesis, 5 μg RNA was used. Indirect labeling was performed with the CyScribe first-strand cDNA labeling kit (Amersham, United Kingdom) according to the manufacturer's protocol. The cDNA samples were labeled with cyanine 3 and cyanine 5. After labeling, the cDNA concentration and the labeling-efficiency were determined using the ND-1000 spectrophotometer. Each microarray was hybridized with 0.5 μg labeled Cy3 and Cy5 cDNA. A total of 12 custom designed microarrays (Agilent Technologies) were used for the comparison between the *L. plantarum *harboring pNZ7021 and pNZ7026 in continuous culture. Both strains were also cultivated in pH regulated batch culture on CDM with and without *p*ABA; for this experiment 21 microarrays were used. Microarrays were hybridized and washed according to the manufactures protocol. Slides were scanned with a ScanArray Express scanner (Perkin-Elmer), using a 10-μm resolution. Images were analyzed with the ImaGene 4.2 software (BioDiscovery, Inc.). Raw data are deposited on GEO under accession number GSM226923 till GSM226943 for the batch experiment microarrays and GSM239110 till GSM239121 for the continuous culture experiment, respectively.

The fraction of folate mRNAs as part of the total mRNA pool was determined as follows. First the signals from the control spots, which are needed for validation purposes, on the custom designed Agilent DNA-micro-arrays were removed from the raw data set, assuring that only 8012 *L. plantarum *probes, representing 2792 genes (91.5% of the genome), were measured. From each probe the intensity of the foreground-signal and background-signal was measured separately for Cy3 and Cy5 signals. The pure probe signal was determined by subtracting the background from the foreground signal. Total signal was determined by summing the raw probe signal of all 8012 probes, the folate signal was determined by adding-up the raw probe signals of the 18 folate probes.

Microarray hybridization schemes were made for the continuous culture experiment and the batch experiment performed in the presence and absence of *p*ABA. The continuous culture scheme consisted of a loop design with 12 microarrays with the following samples hybridized on one array and labeled with Cy3 and Cy5, respectively: C1 and F1, F1 and C3, C3 and F2, F2 and C2, C2 and F3, and F3 and C1, C1 and C2, F2 and F1, C4 and F4, C2 and C4, F4 and F1, and F4 and C3. Here, C1, C2, C3, and C4 represent fourfold biological replicates from *L. plantarum *harboring pNZ7021. F1, F2, F3, and F4 represent fourfold biological replicates of *L. plantarum *harboring pNZ7026. The experimental scheme for the batch experiment performed with and without *p*ABA, consisted of a loop design with 21 microarrays with the following samples hybridized on one array and labeled with Cy3 and Cy5, respectively: C1+P and F1+P, F1+P and C2+P, C2+P and F3+P, F3+P and C3+P, C3+P and F2+P, F2+P and C1+P, C1+P and C2+P, F2+P and F3+P, C1-P and F1-P, F1-P and C2-P, C2-P and F3-P, F3-P and C3-P, C3-P and F2-P, F2-P and C1-P, C1-P and C2-P, F2-P and F3-P, C3-P and F1+P, F2+P and C1-P, C2+P and F3-P, F1-P and C3+P, and F2+P and F1-P. Here, C1+P, C2+P, C3+P, F1+P, F2+P, and F3+P represent threefold biological replicates of the *L. plantarum *harboring pNZ7021 and pNZ7026, respectively, when grown in batch in the presence of *p*ABA. The C1-P, C2-P, C3-P, F1-P, F2-P, and F3-P, represent the *L. plantarum *harboring pNZ7021 and pNZ7026, respectively, when grown in batch in the absence of *p*ABA.

Microarray data were analyzed as described previously [52]. The statistical significance of differences was calculated from variation in biological replicates, using the eBayes function in Limma (cross-probe variance estimation) and Holmes determination of significance. Only genes with a log2 ratio of -1 and +1 and a Holmes value less than 0.1 were considered significant.

The microarray platform and microarray data are available at the Gene Expression Omnibus http://www.ncbi.nlm.nih.gov/geo under the accession numbers given above.

### Metabolome analysis

The complete metabolome of *L. plantarum *WCFS1 harboring pNZ7021 and pNZ7026 from continuous cultivation, in three independent replicates, was quenched using the sodium chloride-method as described previously [53]. After dissolving in water, the intracellular metabolites were profiled in an untargeted manner on a reversed phase HPLC-MS system with a high resolution accurate mass detector (QTOF Ultima MS) as described before [54]. A Synergi Hydro-RP column, 250 × 2.0 mm and 4 μm pore size (Phenomenex, USA), and a gradient of 0 to 35% acetonitrile in water (acidified with 0.1% formic acid) during 45 min were used to separate the metabolites. Full scan accurate mass data (m/z 80-1500) were collected in both positive and negative electrospray ionization mode, using leucine enkephalin as a lock mass. Hereafter the mass signals exceeding three times the local noise were extracted, and mass profiles of both strains were compared using MetAlign™ software [54-56]. This program is designed for determining significant differences in the relative abundance of mass signals originating from metabolites. Based on their accurate masses and MS/MS fragmentation patterns, metabolites have been annotated by using the PubChem DB metabolite database http://www.ncbi.nlm.nih.gov.

### Determining the relative copy number of the pNZ derived plasmids

The relative copy number was determined by quantitative PCR (qPCR). One primer-set was designed for the CM resistance gene on the plasmids pNZ7021 and pNZ7026, the other primer set was designed for the tryptophan gene, *trpE*, on the chromosome of *L. plantarum *WCFS1. The primers for the CM gene on the plasmid contain the following sequences, CTTAGTGACAAGGGTGATAAACTCAAA and CAATAACCTAACTCTCCGTCGCTAT, for the forward and reverse primers, respectively. The primer sequences of the tryptophan gene, *trpE*, on the chromosome of *L. plantarum *WCFS1 were as follows: GCTGGCGCGCCTAAGA (forward primer) and GCGGCACCTGCTCATAATG (reverse primer). The primers for the chromosome are used as marker for the chromosomal copy number to which all plasmid copy numbers were compared; this determines the relative copy number. Total DNA fraction was isolated from *L. plantarum *in the stationary phase. Total DNA was isolated from 5 ml of cell pellet using established procedures [43]. For qPCR, 0.2 μg of total DNA was used. The amplification efficiency was determined for: genomic DNA of *L. plantarum *WCFS1, pNZ7021 plasmid DNA and pNZ7026 plasmid DNA, amplification factors ranging from 1.9 to 2.0 were considered to be reliable. Sybr Green (ABI, Cheshire, UK) was used as fluorescent dye for determining the level of amplification. The Critical threshold number (*C_t_*) was determined using ABI Prism 7500 Fast Real-Time PCR system and software. The C_t _value was used to calculate the relative gene copy number (*N*_relative_) for the plasmid copy number in relation to the chromosomal copy number with the formula *N*_relative _= 2^(*C*^*_t_*_,*plasmid*_^-*C*^*_tchromosome_*^)^. *C_t_*_,*plasmid *_is C_t _value for plasmid and *C_t_*_,chromosome _is *C_t _*value for the chromosome. All relative copy number determinations were performed in triplicate.

### RT-qPCR

Cells of *L. plantarum *WCFS1 cultures were quenched using the cold methanol method as described above. RNA was extracted, quantified, and checked for quality as described above. Primers were used to convert specific mRNA molecules into cDNA using a first-strand cDNA synthesis kit (Amersham, United Kingdom). In *L. plantarum *harboring pNZ8148 and pNZ7030 the following primers were used for cDNA synthesis: groES-re(2), pfk-re1, RQPCRfolBS, and RQPCRFPS. In *L. plantarum *harboring pNZ7031 the following primers were used for cDNA synthesis: groES-re(2), pfk-re1, RQPCRfolBAS, and RQPCRfPAS. The sequence of the primers can be found in Table 7. All cDNA samples were diluted 100-fold to allow accurate quantification by qPCR. Sybr Green (ABI, Cheshire, UK) was used as fluorescent dye for determining the level of amplification. For qPCR on *groES*, *pfK*, *folBS*, *folBAS*, *folPS*, and *folPAS *the following primers-sets were used: groES-fo(2) and groES-re(2), pfk-fo1 and pfk-re2, FQPCRfolBS and RQPCRfolBS, FQPCRfolBAS and RQPCRfolBAS, FQPCRFPS and RQPCRFPS, and FQPCRfPAS and RQPCRfPAS, respectively. The Critical threshold number (*C_t_*) was determined using ABI Prism 7500 Fast Real-Time PCR system and software. The C_t _value was used to calculate the relative gene expression (*N*_relative_) using the formula *N*_relative _= 2^((*C*^*_tRF_*^-*C*^*_tRN_*^)-(C^*_tEF_*^-C^*_tEN_*^))^. In this formula, *C_tRF _*and *C_tRN _*represent the *C_t _*value in the reference strain for the folate gene and normalizing gene, respectively. C_tEF _and C_tEN _are the C_t _value for the tested strain for the folate and normalizing gene, respectively.

### SDS-PAGE and protein quantification

Protein was isolated as described previously [57]. To determine the level of protein overexpression, SDS-PAGE was performed as described previously [44]. The level of protein overexpression was quantified using ImageJ http://rsb.info.nih.gov/ij/. The program ImageJ has a package for the conversion of protein bands into peaks, each peak can be quantified by determining the area.

## List of abbreviations used

The abbreviations used are: CDM: Chemically Defined Medium; C_t_: Critical threshold; GTP: Guanosine triphosphate; HPLC: High Performance Liquid Chromatography; LC-MS/MS: Liquid Chromatography-Mass Spectrometry/Mass Spectrometry; Limma: Linear models for microarray data; MAST: Motif Alignment and Search Tool; MEME: Multiple EM for Motif Elicitation; mRNA: messenger RNA; *p*ABA: para-aminobenzoic acid; PCR: polymerase chain reaction; SDS-PAGE: Sodium Dodecyl Sulfate Poly Acrylamide Gel Electrophoreses; RT-qPCR: Reverse Transcriptase quantitative polymerase chain reaction;.

## Competing interests

The authors declare that they have no competing interests.

## Authors' contributions

AW constructed overexpression strain, preformed microarray experiments, QPCR, folate analysis, SDS Page and drafted the manuscript. AEM and MF carried out some of the chemostat cultures for obtaining data for metabolomics and transcriptomics. DM developed the microarrays and helped analyzing the data. RCHdeV performed the differential metabolomics work and analyzed the data. SMJK and ADH performed the pterine analysis. WMdeV and EJS supervised the study and reviewed results. All authors have read and approved the final manuscript.

## References

[B1] Wegkamp A van Oorschot W de Vos WM Smid EJ Characterization of the role of para-aminobenzoic acid biosynthesis in folate production by Lactococcus lactis Appl Environ Microbiol 2007 73 8 2673 2681 10.1128/AEM.02174-06 17308179 PMC1855612

[B2] Burgess C O'Connell-Motherway M Sybesma W Hugenholtz J van Sinderen D Riboflavin production in Lactococcus lactis: potential for in situ production of vitamin-enriched foods Appl Environ Microbiol 2004 70 10 5769 5777 10.1128/AEM.70.10.5769-5777.2004 15466513 PMC522069

[B3] Hols P Kleerebezem M Schanck AN Ferain T Hugenholtz J Delcour J de Vos WM Conversion of Lactococcus lactis from homolactic to homoalanine fermentation through metabolic engineering Nat Biotechnol 1999 17 6 588 592 10.1038/9902 10385325

[B4] Ladero V Ramos A Wiersma A Goffin P Schanck A Kleerebezem M Hugenholtz J Smid EJ Hols P High-level production of the low-calorie sugar sorbitol by Lactobacillus plantarum through metabolic engineering Appl Environ Microbiol 2007 73 6 1864 1872 10.1128/AEM.02304-06 17261519 PMC1828817

[B5] Green JBPN Matthews RG Folate Biosynthesis, reduction, and polyglutamylation, P. 665-673. In F.C. Neidhardt (Ed.), Escherichia Coli and Salmonella 1996 1 Washington DC, USA

[B6] Neale GA Mitchell A Finch LR Formylation of methionyl-transfer ribonucleic acid in Mycoplasma mycoides subsp. mycoides J Bacteriol 1981 146 2 816 818 6163766 10.1128/jb.146.2.816-818.1981 PMC217031

[B7] Stover P Schirch V The metabolic role of leucovorin Trends Biochem Sci 1993 18 3 102 106 10.1016/0968-0004(93)90162-G 8480361

[B8] Baneyx F Recombinant protein expression in Escherichia coli Curr Opin Biotechnol 1999 10 5 411 421 10.1016/S0958-1669(99)00003-8 10508629

[B9] Williams DC Van Frank RM Muth WL Burnett JP Cytoplasmic inclusion bodies in Escherichia coli producing biosynthetic human insulin proteins Science 1982 215 4533 687 689 10.1126/science.7036343 7036343

[B10] Schoner RG Ellis LF Schoner BE Isolation and purification of protein granules from Escherichia coli cells overproducing bovine growth hormone. 1985 Biotechnology 1992 24 349 352 1422038

[B11] Nagata S Taira H Hall A Johnsrud L Streuli M Ecsodi J Boll W Cantell K Weissmann C Synthesis in E. coli of a polypeptide with human leukocyte interferon activity Nature 1980 284 5754 316 320 10.1038/284316a0 6987533

[B12] Gelfand DH Shepard HM O'Farrell PH Polisky B Isolation and characterization of ColE1-derived plasmid copy-number mutant Proc Natl Acad Sci USA 1978 75 12 5869 5873 10.1073/pnas.75.12.5869 104293 PMC393077

[B13] Bentley WM Mirjalili N Andersen DC Davis RH Kompala DS Plasmid-encoded protein: The principal factor in the "Metabolic Burden" associated with recombinant bacteria Niotechnology and Bioengineering 1990 35 668 681 10.1002/bit.260350704 18592563

[B14] Haddadin FT Harcum SW Transcriptome profiles for high-cell-density recombinant and wild-type Escherichia coli Biotechnol Bioeng 2005 90 2 127 153 10.1002/bit.20340 15742388

[B15] Kurland CG Dong H Bacterial growth inhibition by overproduction of protein Mol Microbiol 1996 21 1 1 4 10.1046/j.1365-2958.1996.5901313.x 8843428

[B16] Vescovo MS Torriani Dellaglio F Bottazzi V Basic characteristics, ecology and application of *Lactobacillus plantarum*: a review Ann Microbiol Enzimol 1993 43 261 284

[B17] Kleerebezem M Boekhorst J van Kranenburg R Molenaar D Kuipers OP Leer R Tarchini R Peters SA Sandbrink HM Fiers MW Complete genome sequence of Lactobacillus plantarum WCFS1 Proc Natl Acad Sci USA 2003 100 4 1990 1995 10.1073/pnas.0337704100 12566566 PMC149946

[B18] Wegkamp A de Vos WM Smid EJ Folate overproduction in Lactobacillus plantarum WCFS1 causes methotrexate resistance FEMS microbiology letters 2009 297 2 261 265 10.1111/j.1574-6968.2009.01690.x 19566681

[B19] Vieira E Shaw E The utilization of purines in the biosynthesis of folic acid J Biol Chem 1961 236 2507 2510 13781236

[B20] Siezen R Boekhorst J Muscariello L Molenaar D Renckens B Kleerebezem M Lactobacillus plantarum gene clusters encoding putative cell-surface protein complexes for carbohydrate utilization are conserved in specific gram-positive bacteria BMC Genomics 2006 7 126 10.1186/1471-2164-7-126 16723015 PMC1534035

[B21] Teusink B Wiersma A Molenaar D Francke C de Vos WM Siezen RJ Smid EJ Analysis of growth of Lactobacillus plantarum WCFS1 on a complex medium using a genome-scale metabolic model J Biol Chem 2006 281 52 40041 40048 10.1074/jbc.M606263200 17062565

[B22] Petranovic D Guedon E Sperandio B Delorme C Ehrlich D Renault P Intracellular effectors regulating the activity of the Lactococcus lactis CodY pleiotropic transcription regulator Mol Microbiol 2004 53 2 613 621 10.1111/j.1365-2958.2004.04136.x 15228538

[B23] Poolman B Smid EJ Veldkamp H Konings WN Bioenergetic consequences of lactose starvation for continuously cultured Streptococcus cremoris J Bacteriol 1987 169 4 1460 1468 3558320 10.1128/jb.169.4.1460-1468.1987 PMC211968

[B24] Regenberg B Grotkjaer T Winther O Fausboll A Akesson M Bro C Hansen LK Brunak S Nielsen J Growth-rate regulated genes have profound impact on interpretation of transcriptome profiling in Saccharomyces cerevisiae Genome biology 2006 7 11 R107 10.1186/gb-2006-7-11-r107 17105650 PMC1794586

[B25] Nahku R Valgepea K Lahtvee PJ Erm S Abner K Adamberg K Vilu R Specific growth rate dependent transcriptome profiling of Escherichia coli K12 MG1655 in accelerostat cultures Journal of biotechnology 2010 145 1 60 65 10.1016/j.jbiotec.2009.10.007 19861135

[B26] Nierlich DP Regulation of bacterial growth, RNA, and protein synthesis Annual review of microbiology 1978 32 393 432 10.1146/annurev.mi.32.100178.002141 360971

[B27] Bremer H Dennis PP Modulation of chemical composition and other parameters of the cell by growth rate 1987 Washington DC: ASM 10.1128/ecosal.5.2.3 26443740

[B28] Snoep JL Yomano LP Westerhoff HV Ingram LO Protein burden in *Zymomonas mobilis*: negative flux and growth control due to overproduction of glycolytic enzymes Microb 1995 141 8

[B29] Gong M Gong F Yanofsky C Overexpression of tnaC of Escherichia coli inhibits growth by depleting tRNA2Pro availability J Bacteriol 2006 188 5 1892 1898 10.1128/JB.188.5.1892-1898.2006 16484200 PMC1426567

[B30] Sybesma W Starrenburg M Tijsseling L Hoefnagel MH Hugenholtz J Effects of cultivation conditions on folate production by lactic acid bacteria Appl Environ Microbiol 2003 69 8 4542 4548 10.1128/AEM.69.8.4542-4548.2003 12902240 PMC169137

[B31] Cooper RG Chen TS King MA Thermal destruction of folacin in microwave and conventional heating J Am Diet Assoc 1978 73 4 406 410 701671

[B32] Nguyen MT Indrawati Hendrickx M Model studies on the stability of folic acid and 5-methyltetrahydrofolic acid degradation during thermal treatment in combination with high hydrostatic pressure J Agric Food Chem 2003 51 11 3352 3357 10.1021/jf026234e 12744666

[B33] Nguyen MT Indrawati Van Loey A Hendrickx M Effect of pH on temperature stability of folates Commun Agric Appl Biol Sci 2004 69 2 203 206 15560222

[B34] Blakley RL The biochemistry of folic acid and related pteridines 1969 Amsterdam and Londen: North Holland

[B35] Caldon CE Yoong P March PE Evolution of a molecular switch: universal bacterial GTPases regulate ribosome function Mol Microbiol 2001 41 2 289 297 10.1046/j.1365-2958.2001.02536.x 11489118

[B36] Kilstrup M Hammer K Ruhdal Jensen P Martinussen J Nucleotide metabolism and its control in lactic acid bacteria FEMS Microbiol Rev 2005 29 3 555 590 10.1016/j.fmrre.2005.04.006 15935511

[B37] Lopez JM GTP pool expansion is necessary for the growth rate increase occurring in Bacillus subtilis after amino acids shift-up Arch Microbiol 1982 131 3 247 251 10.1007/BF00405887 6808962

[B38] Martinussen J Hammer K Powerful methods to establish chromosomal markers in *Lactococcus lactis *- an analysis of pyrimidine salvage pathway mutants obtained by positive selections Microbiology (UK) 1995 141 1883 1890 10.1099/13500872-141-8-1883 20369419

[B39] Teusink B van Enckevort FH Francke C Wiersma A Wegkamp A Smid EJ Siezen RJ In silico reconstruction of the metabolic pathways of Lactobacillus plantarum: comparing predictions of nutrient requirements with those from growth experiments Appl Environ Microbiol 2005 71 11 7253 7262 10.1128/AEM.71.11.7253-7262.2005 16269766 PMC1287688

[B40] Otto RB ten Brink H Veldkamp H Konings WN The relation between growth rate and electrochemical proton gradient of Streptococcus cremoris FEMS Microbiology Letter 1983 16 69 74 10.1111/j.1574-6968.1983.tb00261.x

[B41] Poolman B Konings WN Relation of growth of Streptococcus lactis and Streptococcus cremoris to amino acid transport J Bacteriol 1988 170 2 700 707 3123462 10.1128/jb.170.2.700-707.1988 PMC210711

[B42] Terzaghi BE Sandine WE Improved Medium for Lactic Streptococci and Their Bacteriophages Appl Microbiol 1975 29 6 807 813 16350018 10.1128/am.29.6.807-813.1975 PMC187084

[B43] Bernard N Ferain T Garmyn D Hols P Delcour J Cloning of the D-lactate dehydrogenase gene from Lactobacillus delbrueckii subsp. bulgaricus by complementation in Escherichia coli FEBS Lett 1991 290 1-2 61 64 10.1016/0014-5793(91)81226-X 1915894

[B44] Kuipers OP de Ruyter PG Kleerebezem M de Vos WM Quorum sensing-controlled gene expression in lactic acid bacteria J Biotechnol 1998 64 15 21 10.1016/S0168-1656(98)00100-X

[B45] de Vos WM Vos P de Haard H Boerrigter I Cloning and expression of the Lactococcus lactis subsp. cremoris SK11 gene encoding an extracellular serine proteinase Gene 1989 85 1 169 176 10.1016/0378-1119(89)90477-0 2515994

[B46] Pavan S Hols P Delcour J Geoffroy MC Grangette C Kleerebezem M Mercenier A Adaptation of the nisin-controlled expression system in Lactobacillus plantarum: a tool to study in vivo biological effects Appl Environ Microbiol 2000 66 10 4427 4432 10.1128/AEM.66.10.4427-4432.2000 11010894 PMC92320

[B47] Josson K Scheirlinck T Michiels F Platteeuw C Stanssens P Joos H Dhaese P Zabeau M Mahillon J Characterization of a gram-positive broad-host-range plasmid isolated from Lactobacillus hilgardii Plasmid 1989 21 1 9 20 10.1016/0147-619X(89)90082-6 2727147

[B48] Sybesma W Starrenburg M Kleerebezem M Mierau I de Vos WM Hugenholtz J Increased production of folate by metabolic engineering of Lactococcus lactis Appl Environ Microbiol 2003 69 6 3069 3076 10.1128/AEM.69.6.3069-3076.2003 12788700 PMC161528

[B49] Horne DW Patterson D Lactobacillus casei microbiological assay of folic acid derivatives in 96-well microtiter plates Clin Chem 1988 34 11 2357 2359 3141087

[B50] Klaus SM Wegkamp A Sybesma W Hugenholtz J Gregory JF Hanson AD A nudix enzyme removes pyrophosphate from dihydroneopterin triphosphate in the folate synthesis pathway of bacteria and plants J Biol Chem 2005 280 7 5274 5280 10.1074/jbc.M413759200 15611104

[B51] Pieterse B Jellema RH van der Werf MJ Quenching of microbial samples for increased reliability of microarray data J Microbiol Methods 2006 64 2 207 216 10.1016/j.mimet.2005.04.035 15982764

[B52] Saulnier DM Molenaar D de Vos WM Gibson GR Kolida S Identification of prebiotic fructooligosaccharide metabolism in Lactobacillus plantarum WCFS1 through microarrays Appl Environ Microbiol 2007 73 6 1753 1765 10.1128/AEM.01151-06 17261521 PMC1828832

[B53] Faijes M Mars AE Smid EJ Comparison of quenching and extraction methodologies for metabolome analysis of Lactobacillus plantarum Microb Cell Fact 2007 6 1 27 10.1186/1475-2859-6-27 17708760 PMC2031893

[B54] De Vos RC Moco S Lommen A Keurentjes JJ Bino RJ Hall RD Untargeted large-scale plant metabolomics using liquid chromatography coupled to mass spectrometry Nat Protoc 2007 2 4 778 791 10.1038/nprot.2007.95 17446877

[B55] America AH Cordewener JH van Geffen MH Lommen A Vissers JP Bino RJ Hall RD Alignment and statistical difference analysis of complex peptide data sets generated by multidimensional LC-MS Proteomics 2006 6 2 641 653 10.1002/pmic.200500034 16372275

[B56] Tikunov Y Lommen A de Vos CH Verhoeven HA Bino RJ Hall RD Bovy AG A novel approach for nontargeted data analysis for metabolomics. Large-scale profiling of tomato fruit volatiles Plant Physiol 2005 139 3 1125 1137 10.1104/pp.105.068130 16286451 PMC1283752

[B57] Boels IC Kleerebezem M de Vos WM Engineering of carbon distribution between glycolysis and sugar nucleotide biosynthesis in Lactococcus lactis Appl Environ Microbiol 2003 69 2 1129 1135 10.1128/AEM.69.2.1129-1135.2003 12571039 PMC143634

